# Unmet needs of patients with chronic obstructive pulmonary disease (COPD): a qualitative study on patients and doctors

**DOI:** 10.1186/1471-2296-15-67

**Published:** 2014-04-16

**Authors:** Stalia SL Wong, Nurdiana Abdullah, Adina Abdullah, Su-May Liew, Siew-Mooi Ching, Ee-Ming Khoo, Moyez Jiwa, Yook-Chin Chia

**Affiliations:** 1 Department of Primary Care Medicine, University of Malaya Primary Care Research Group (UMPCRG), Faculty of Medicine, University of Malaya, Kuala Lumpur 50603, Malaysia; 2 Department of Family Medicine, Faculty of Medicine and Health Sciences, Universiti Putra Malaysia, Serdang, Selangor 43400, Malaysia; 3 Curtin Health Innovation Research Institute, Faculty of Health Sciences, Curtin University, GPO Box U1987, Western Australia, Perth 6845, Australia

**Keywords:** COPD, Qualitative, Self-management, Knowledge, Quality of life, Needs

## Abstract

**Background:**

Chronic Obstructive Pulmonary Disease (COPD) is a chronic disease with repeated exacerbations resulting in gradual debilitation. The quality of life has been shown to be poor in patients with COPD despite efforts to improve self-management. However, the evidence on the benefit of self-management in COPD is conflicting. Whether this could be due to other unmet needs of patients have not been investigated. Therefore, we aimed to explore unmet needs of patients from both patients and doctors managing COPD.

**Methods:**

We conducted a qualitative study with doctors and patients in Malaysia. We used convenience sampling to recruit patients until data saturation. Eighteen patients and eighteen doctors consented and were interviewed using a semi-structured interview guide. The interviews were audio-recorded, transcribed verbatim and checked by the interviewers. Data were analysed using a thematic approach.

**Results:**

The themes were similar for both the patients and doctors. Three main themes emerged: knowledge and awareness of COPD, psychosocial and physical impact of COPD and the utility of self-management. Knowledge about COPD was generally poor. Patients were not familiar with the term chronic obstructive pulmonary disease or COPD. The word ‘asthma’ was used synonymously with COPD by both patients and doctors. Most patients experienced difficulties in their psychosocial and physical functions such as breathlessness, fear and helplessness. Most patients were not confident in self-managing their illness and prefer a more passive role with doctors directing their care.

**Conclusions:**

In conclusion, our study showed that knowledge of COPD is generally poor. There was mislabelling of COPD as asthma by both patients and physicians. This could have resulted in the lack of understanding of treatment options, outcomes, and prognosis of COPD. The misconception that cough due to COPD was contagious, and breathlessness that resulted from COPD, had important physical and psychosocial impact, and could lead to social isolation. Most patients and physicians did not favour self-management approaches, suggesting innovations based on self-management may be of limited benefit.

## Background

Chronic Obstructive Pulmonary Disease (COPD) is a common cause of morbidity and mortality throughout the world. It is the third leading global cause of death in 2010 after ischaemic heart disease and stroke [1]. The Malaysian prevalence of moderate to severe COPD was estimated to be 4.7% [2]. COPD is among the top five leading cause of years lived with disability (YLDs) in Malaysia and is the sixth leading cause of loss of disability adjusted life years (DALYs) in 2010 [3].

COPD is a chronic disease with repeated exacerbations resulting in gradual debilitation. The quality of life is reduced in these patients [4,5]. There has been limited progress in the management of the disease and as such, greater emphasis should be given to address the quality of life for these patients. In a study of outpatients with COPD, 46% had significant breathlessness and 39% graded their quality of life as low [5]. Poorer quality of life was also associated with greater severity of the disease [4].

There have been studies looking at self-management in improving the quality of life in patients with COPD. Bourbeau et al has defined self-management as any formalized patient education program aimed at teaching skills needed to carry out specific medical regimens specific to the disease and guide behaviour change for patients to control their disease and improve their well-being [6]. A Cochrane review of self-management education showed that there was a slight reduction in dyspnoea and also a statistically significant improvement in quality of life which did not reach clinical relevance [7]. There was no significant reduction in the number of exacerbations, emergency department visits, lung function, exercise capacity and days lost from work [7]. A systematic review by Bentsen et al, however, had shown that self-management intervention tend to improve the total health status of COPD patients [8]. Another study reported that self-treatment of exacerbations was cost-effective and leads to fewer exacerbation days [9].

In view of these conflicting findings, we aimed to investigate unmet needs of patients with COPD that could potentially improve their quality of life. We specifically set out to explore the perceived needs and expectations of patients with regards to their lifestyle, management and adaptation towards the disease.

## Methods

Qualitative method was chosen in order to obtain an in-depth exploration of the views and ideas of patients’ perceived needs in managing COPD. The study was carried out on patients and doctors from a tertiary hospital located in an urban suburb of Kuala Lumpur, Malaysia in 2012. Focus group discussions (FGDs) were conducted separately for patients and doctors. All the FGDs were conducted at the hospital except for one which was conducted at a community health clinic. Ethics approval from the Medical Ethics Committee, University Malaya Medical Centre was obtained (MEC Ref no: 896.11).

### Sample and data collection

#### Patient participants

Participants with a diagnosis of COPD confirmed by spirometry were recruited from the chest clinic of the hospital using convenience sampling by a research assistant. Out of a total of 30 patients invited, 12 were not interested and refused to participate in the study. Details of the study were provided to the participants and informed consent was obtained. The focus group discussion was led by a researcher who was not directly involved in the management of the patients. A nominal payment equivalent to USD15 was offered to the participants to cover travel costs.

#### Doctor participants

Doctors who manage patients with COPD were recruited from the hospital chest clinic and primary care clinics from the surrounding area using convenience sampling and were invited to participate in focus group discussions by one of the researchers. A total of 26 doctors were invited and 8 were not interested to participate. Details of the study were provided to the participants and informed consent obtained.

#### Focus group discussion

The FGDs were conducted using a semi-structured topic guide. The discussions among the patient participants explored their most worrying problems since diagnosed with COPD, their source of knowledge regarding COPD, their needs and expectations, their perception on managing their own disease and their opinion on how far they would like to be involved in managing their disease. Similarly, the discussions among the doctor participants looked into the problems experienced by COPD patients, the patients’ perceived needs and expectations, patients’ source of knowledge, the doctors’ perception on self-management of COPD and how far should COPD patients be involved in managing their disease. Self-management strategies included self-adjustment of medication/inhaler dose, self-administering short courses of oral steroids and antibiotics during exacerbations, domiciliary nebulizer use and carrying out rehabilitation exercises at home. The guide was based on a theoretical framework devised by Golla et al [10]. The topic guides are available on request from the authors. Each FGD lasted between one to two hours. During the discussions, the facilitators repeatedly summarized information obtained to check for accuracy with participants.

A total of 8 FGDs (4 with a total of 18 patients and 4 with a total of 18 doctors) were conducted. For the FGDs with patients, after the third FGD, there was no further new information emerged and data saturation was deemed to have been achieved. Similarly, for FGDs with the doctors, data saturation was reached at the second FGD. The FGDs were audio-recorded and transcribed verbatim. All identifying information was removed in the transcripts to maintain anonymity. Each transcript was then checked for accuracy by a separate researcher or assistant listening to the audio records.Transcripts were kept secure in a locked file cabinet for data safe keeping and confidentiality.

### Analysis

Transcripts of focus groups were coded independently by 2 researchers (SWSL, NA) after familiarising with the transcripts. There were few discrepancies in coding, which were resolved by discussions and consensus was made. Thematic analysis was used to identify themes. The emerging themes were then discussed by all members of the research team. This resulted in the final themes that could be applied to all data. Relevant quotations were identified and selected from the transcripts to highlight the themes.

## Results

There were 18 patient participants and 18 doctors, which comprised of 3 respiratory physicians and 15 primary care doctors. All patient participants were males and ex-smokers with their age ranged from 52 years to 89 years (mean: 72.3 years). Among the patient participants, there was 44.4% Chinese, 38.9% Malays and 16.7% Indians; 44.4% had primary school education and another 44.4% had secondary school education. The mean value of the FEV1/FVC for participants on treatment was 59% (SD: 12.8%).

For the doctor participants, there was 38.9% Malays, 27.8% Indians, 22.2% Chinese and 11.1% other Asian ethnic groups. A total of 13 (72.2%) doctor participants were females. The experience in managing COPD patients ranged from 7 to 35 years (mean: 16.8 years).

The themes that emerged were similar in both the patient and doctor groups. There were three main themes: a) knowledge and awareness of COPD, b) psychosocial and physical impact of COPD and c) the utility of self-management.

### Knowledge and awareness of COPD

#### Terminology

Terminology was identified as an important issue. Patient participants were not familiar with the term chronic obstructive pulmonary disease or COPD. The term ‘asthma’ was used synonymously with COPD by both patients and doctors. One of the patients used the term ‘asthma’ throughout the focus group discussion to refer to his disease. He recounted how the doctor called him an ‘asthmatic’ during an exacerbation.

P7

“They admitted me for one day; they checked and tested me up. They said you have asthma.”

Another patient found it difficult to remember what COPD stands for.

P4

“Too long, I don’t know, I can’t remember.”

Doctors also acknowledged the difficulty with the term COPD and the misuse of asthma by both patients and doctors. The labelling of patients with COPD as being ‘asthmatic’ led to difficulties in improving patient knowledge and treatment. COPD was seen as a complex term and patients’ understanding of COPD is poorer compared to other conditions such as diabetes and hypertension. A doctor commented that the term “asthma” was used because it was more familiar to patients.

D5

“I think it is not so common like diabetes, hypertension; you ask anybody, they know, but COPD very few and then, you know they don’t know what it means, what are the complications, many they don’t know, they are not well educated.”

D17

“We may use the English word to say, we have to say 4 letters, C, O, P, D, whereas when you say asthma, they know it’s asthma already, even the non-English speaker, they know asthma, what it is, but when you want to say the word C, O, P, D, err.. you use the word ‘breathlessness’ or whatever, it’s just a description of symptoms, you cannot convey to them this is another disease. How very long - chronic, obstructive…because the word used is not a layman term, asthma is very easy for them.”

D 9

“And a lot of people, they feel that COPD is …“oh, I’m having asthma”, and then “why don’t you just give medication for asthma?”

D 15

“A lot of them, they don’t even know what is COPD. If symptoms are mainly breathlessness, then it’s ‘asthma’.”

#### Knowledge of disease

All patient participants were smokers. Patients generally had poor knowledge of the aetiology of the disease prior to being diagnosed. None was aware that smoking causes COPD. However, most were aware that smoking causes lung cancer.

P1

“I know smoking is bad – I thought it caused cancer, although I never heard of COPD….Cancer, cancer, cancer, that’s all I know.”

Patients commented on the lack of patient information and education regarding COPD. The majority of their information on COPD was obtained from their doctor. Other information sources quoted were the internet, media, books and family members.

P1

“Yes, I know very little about COPD, because when you see the doctor in-charge, they just tick-tick-tick, jot and tick. And I read the pamphlets, what those inhalers are for but there is very little.”

Self-management knowledge was generally poor.

P4

“Don’t know how to do it, we don’t know. The thing is, we don’t know how to do it.”

P2

“I don’t know, I’m better consult the doctor. Scared something wrong…”

Most patients referred to their inhaler by the colours (purple for prophylactic medication and blue for the bronchodilator). Only two could name the medication used. Most patients were unaware of the need for influenza and pneumococcal vaccination. Doctors also agreed that patients had poor knowledge about their disease or its management. There was even one patient who believed that his COPD would get better with time.

P14

“I hope the doctor gives good medication to cure our disease. I feel that it may take four to five years for the COPD to disappear. I have only had the disease for one year.”

### Psychosocial and physical impact of COPD

Most patients experienced difficulties in their psychosocial and physical functions which affected their lifestyle.

#### Psychosocial limitations

Breathlessness was associated with a negative psychosocial impact. Patients were fearful at being left alone. They were worried that help could not arrive in time. One of the participants mentioned that he felt as if he might die during an attack.

P4

“I am afraid to stay at home alone. If anything happens, nobody can help me. I am afraid that I will be ‘out-of-order’ (might die).”

Some participants experienced a loss of intimacy. One patient expressed difficulty in performing sexual intercourse. A few participants slept separately from their partner because their partner wanted to have the air conditioner or fan on through the night.

P7

“The problem is my wife, she wants the air-conditioning on. So I go to sleep in a different room.”

Others experienced loss of social interaction and bonding. Their breathlessness limited their participation in family gatherings. Some avoided crowded places as they believed this would aggravate their condition. One patient felt that he needed air to breathe if another person is at close proximity to him.

P5

“Even someone come near me, I’ll push him. Because no air to breathe. I find that, if you come near me, I don’t have air to breathe, you know. So, I push my wife away.”

The cough was also viewed by family member as contagious.

P7

*“Especially smaller children - we don’t get too near them nowadays. Sometimes the parent would say ‘Oh! The cough may be from you! …Contagious. Don’t get near them.’ Yes, change lifestyle like we have to have a separate cup set - don’t eat together*.”

Patients reported experiencing more stress and being emotional. Some felt the disease were self-inflicted and expressed resignation at their fate.

P18

“We are the ones that are bad. We do not want to stop smoking – nobody’s fault but our own.”

P4

“Because of our smoking, we feel regret. But what can we do? We have already smoked so what can we do.”

Some doctors talked about how patients do not express their psychosocial problems. But others spoke about how patients were not coping and how disease progression and symptoms led to frustration.

D14

“The main thing usually, he would present with clinical, the emotional part sometimes are not really expressed, because, sometimes we don’t have time to explore.”

“I must say that, actually, they are not coping, they are crying for help but they don’t know how to ask for help.”

D12

“Because the COPD is not controlled, then the hypertension and also heart disease keep on happening, so, this patient got frustrated.”

#### Physical impact

Physical function was reported to be severely limited by breathlessness. This included activities of daily living such as talking, eating and ambulating. Many patients limited the amount of food eaten as large meals made them feel breathless. Patients expressed feelings of loss of independence and helplessness resulting in the need for help by caregivers.

P8

“We have to limit our food consumption. If we are too full, it affects our breathing and causes difficulty moving about.”

P4

“Now the situation is different, I have difficulty bathing. I cannot scoop the water, I cannot apply the soap. I feel suffocated, like dying. I do not lock my bathroom door as I am afraid that nobody can help me. I cannot dry myself with the towel. It is tiring.”

P 18

“There is one thing which I regret, I cannot go travelling. I cannot go anywhere as I cannot walk long distances. If my wife is very good and goes to survey first to see if there is a wheelchair, then I’ll go.”

Patients also practiced food restrictions. Many believed that cold beverages and certain fruits aggravated their COPD. Doctors noted that some patients ate honey, ginger or lime to help with their symptoms.

P9

“Cannot eat watermelon. We will be breathless. The white watermelon, I avoid. Iced water, cannot take.”

D1

“Some of them, they do use home remedies, traditional like honey; honey and lime and warm water. Some use ginger to help with the cough and all that.”

Accessing healthcare was difficult for many patients. The majority had difficulty reaching the clinic from the car park. Most preferred to take a taxi that could drop them as near as possible to their intended location. One participant proposed that patients with COPD should be given a permit to enable them to use the disabled parking lot.

P7

“Like the disabled, if we can have facilities, for old people, because the other day, I had to come to Emergency, asthma attack. I had to park my car the other block, the other side, walking down, hot sun, I nearly fainted.”

Most participants coped by breaking a physical activity into small successive steps with rest periods in between each task. This impaired their ability to venture on holidays or even to go shopping. The physical impact leads to frustration towards the illness.

P4

“Because I am slow, they walk faster. So, I have to use a wheel chair, right? But my leg is not broken. I only look well but they don’t know that I am half-dead.”

### The utility of self-management

Most of the patients were not confident in self-managing their illness. Only one patient felt confident enough to adjust his own medications. There was a preference for a more passive role with doctors directing their care.

P4

“It is the doctor’s duty. We are not in the medical field - we wouldn’t know.”

P18

“We cannot depend on ourselves. We need someone to treat and give us the medication. That is our routine.”

Some doctors felt that patients are not able to self-manage. Others felt that it might be possible for patients to adjust inhaler dosage but not oral steroids or antibiotics. They reasoned that this was due to patients’ poor knowledge about their disease. One doctor did not support self-management because she believed that patients should come for follow-up to be properly assessed.

D12

“At least when they come to us, we can assess them and see how severe is the disease.”

D1

“I don’t think that our Asian patients are like that from Western countries, so we cannot expect them to self-care. They might abuse antibiotics.”

D16

“They don’t come to that level yet, self-management, I think, that one, your level of education, motivation must be very high.”

An additional theme that arose from the doctors’ focus group discussions were the challenges in managing patients with COPD. These were with regards to the limited resources in the primary care clinics such as the limited access to diagnostic equipment, more expensive medications, health educational material and rehabilitation services.

D15

“In our setting, we don’t have the spirometry facilities. So, most of the time, we will diagnose by history, risk factor and also clinical symptom mainly, but for those patient when not sure about asthma or COPD, then, we can send for spirometry in nearby hospital.”

“At the moment, I think for COPD, we don’t really have COPD pamphlet or leaflet.”

D6

“I don’t think our medication is enough especially in health clinics, where our resources are very limited. Most of them, we just give them whatever we have which is inadequate for theirtreatment actually. But when we refer to secondary care and they don’t want to go, then it’s very difficult.”

## Discussion

The key finding of this study was the lack of knowledge on COPD, which was the greatest unmet need faced by patients. This is reflected by three main areas: 1. the lack of knowledge on the causes of COPD; 2. the mislabelling of COPD as asthma; 3. the misconception that cough due to COPD was contagious.

Firstly, it is surprising that almost all patients in this study did not know that smoking was a cause of COPD until informed at the time of diagnosis. In spite of not knowing of the link between smoking and COPD, they were very aware that smoking causes lung cancer which is similar with another study [11]. However, this did not stop them from smoking. This paradox may be due to perceived lack of benefit in quitting smoking. It has been shown that smokers who perceived their lifetime risk of having lung cancer is high whether they continued or stop smoking were less likely to see the benefits of quitting smoking [12]. Another study among smokers showed that 99% knew smoking causes lung cancer, however, only 63.5% reported that significant disability could result from smoking [13]. Hence, the link between smoking and COPD which may lead to significant disability and reduced quality of life should be made more widely known as this may deter people from smoking.

Secondly, the mislabelling of COPD as asthma by both patients and doctors was frequent. This caused problems in patients’ expectations of management and prognosis as asthma is considered to be reversible. Sheridan et al also found that COPD is frequently confused with asthma and that this led to a perception that COPD is a reversible condition [14]. We had postulated that the confusion of terminology in our study was due to there being no terms for COPD in local languages. The study by Sheridan was conducted in three different languages and that may have resulted in the similar findings [14]. Part of the reason for this mislabelling is the lack of a linguistic equivalent for COPD in the local language. Asthma, a more prevalent condition, is referred to as ‘lelah’ meaning breathlessness, which is also a common presenting complaint in COPD. This is similar as described in another Asian country, whereby; a term in the local language which means ‘cough and dyspsnoea’ is more familiar to the population [15]. Perhaps, a clearer explanation or new terminology in the local language would better differentiate these two conditions. This is important for better patient understanding of the illness, its aetiology and disease management and prevention. Another contributing factor to mislabelling is the difficulty in making a definitive diagnosis of COPD. Most primary care clinics in Malaysia do not have spirometry equipment and referral to hospital is required for confirmation. These findings indicate the importance of providing access to spirometry for an accurate diagnosis to be made. This will allow patients to have a better understanding of their disease.

Thirdly, social interaction was affected by misinformed beliefs such as the perception of the cough being contagious due to the high prevalence of pulmonary tuberculosis in our setting. Clarification of this misconception could improve family interaction. In terms of the psychosocial and physical impact of the disease, the predominant finding was of fear. Patients were afraid of being left on their own, of being helpless and incapable of coping without the help of others. This led to the progressive loss of independence. These findings are consistent with those of other studies [16,17].

A new finding was the perceived association between large meals and breathlessness. This impaired patients’ satisfaction and enjoyment particularly at family gatherings. As far as we know, this finding has not been reported in the medical literature. It may be explained by splinting of the diaphragm by stomach contents or even possibly gastro-oesophageal reflux. This should be explored further by physiological studies.

Both patients and doctors were against the adoption of self-management strategies. This is contrary to recommendations for the management of COPD by many studies and guidelines [18-20]. However, another study has similarly shown that self-management skills were not rated as important by patients [21]. Furthermore, the psychosocial impact of their disease such as fear limited their ability to manage their own symptoms [22]. A lack of knowledge may also contribute to their dependence on doctors and health care providers [23]. COPD may be a condition where self-management approaches differ according to the severity of patients’ illness. Although many of the patients expressed reluctance in self-management, one patient in this study did self-manage his COPD. An increase in knowledge and awareness of COPD may lead to better patient empowerment and self-efficacy. This would require further studies for verification. In reality, patients have to conduct self-management daily and it is not feasible for physicians to provide all of the management needs that patients have during their day-to-day lives. Therefore, self-management remains an aspect of overall COPD care. However, it should not be the only focus and future interventions should also examine ways to improve access to health care.

### Limitations and strengths of the study

All patient participants were male. This is because more than 94% of COPD patients in the chest clinic were male. There may be differences in the views of female patients and the sampling strategy may not have included the whole spectrum of patients. Also, the recruitment of patients was from a chest clinic, where patients are likely to have more severe disease. For these reasons and because this is a qualitative study, the results are not generalisable to other populations.

The strength of this study is that it included the local doctors’ views, which were not studied previously. The collection of data in both patients and doctors allowed us to triangulate the results. As far as we know, this is the only study that has examined patients’ experiences of COPD from the doctors’ perspective.

## Conclusions

In conclusion, our study showed that knowledge of COPD is generally poor. There was mislabelling of COPD as asthma by both patients and physicians. This could have resulted in a lack of understanding of treatment options, outcomes, and prognosis of COPD. The misconception that cough due to COPD was contagious, and breathlessness that resulted from COPD, had important physical and psychological impact, which could lead to social isolation. Most patients and physicians did not favour self-management approaches, suggesting innovations based on self-management may be of limited benefit.

## Abbreviations

COPD: Chronic obstructive pulmonary disease; FGD: Focus group discussion; YLDs: Years lived with disability; DALYs: Disability adjusted life years; FEV1: Forced expiratory volume in one second; FVC: Forced vital capacity.

## Competing interests

The authors declare that they have no competing interests.

## Authors’ contributions

CYC, SWSL, MJ contributed in the study conception and design. SWSL conducted the focus group discussions among patient participants and NA conducted the focus group discussions among doctor participants. SWSL and NA performed the qualitative analysis. All authors contributed in the discussions of the findings. SWSL, NA, LSM, AA, CSM and CYC drafted the manuscript. MJ, CYC and KEM made critical comments and revisions to the manuscript. All authors read and approved the final manuscript.

## Pre-publication history

The pre-publication history for this paper can be accessed here:

http://www.biomedcentral.com/1471-2296/15/67/prepub
